# Rare Complication of Botox Injection: A Case Report

**DOI:** 10.29252/wjps.8.1.116

**Published:** 2019-01

**Authors:** Abdolreza Rouientan, Hamidreza Alizadeh Otaghvar, Hossein Mahmoudvand, Adnan Tizmaghz

**Affiliations:** 1 Department of Plastic and Reconstructive Surgery, Shahid Beheshti University of Medical Sciences, Tehran, Iran;; 2 Trauma and Injury Research Center, Iran University of Medical Sciences, Tehran, Iran;; 3 Department of Surgery, Iran University of Medical Science, Tehran, Iran

**Keywords:** Botulinum, Toxin, Botox, Complication, Iran

## Abstract

Botulinum toxin (BTX) is also well-known as Botox is produced by a gram-positive anaerobic bacterium called *Clostridium botulinum.* Generally, clinical manifestations of BTX can be observed after consumption of contaminated food, from colonization of the infant gastrointestinal tract, as well as following the infection of the wound to this bacterium. There are seven types of this neurotoxin labeled as A, B, C (C1, C2), D, E, and F. Human botulinum is caused by types A, B, E and rarely F. The most common clinical symptoms of BTX in cosmetic goals are cervical dystonia, severe primary axillary hyperhidrosis, strabismus, neurogenic detrusor over-activity, chronic migraine, upper limb spasticity and blepharospasm. Botox has a wide range of therapeutic uses and occasionally patients receiving this treatment may experience botulism symptom including local and even distant and autonomic symptoms. Despite the efficacies of Botox in treatment of myriad neurologic and cosmetic conditions, it may carry some risk of sever adverse effects which may be the result of local or systemic spreading of the drug. Our patient was a 22 years old man who received Botox for axillary hyperhidrosis after two weeks, when most of generalized complications of botulinum toxin appeared. This case was introduced for being aware of dangerous complication of Botox. Pyridostigmine could relieve symptoms of the patient.

## INTRODUCTION

The German physician, Justinus Kerner (1786-1862) was the first who developed the idea of a possible therapeutic use of botulinum toxin.^1^ In 1973, for the first time, Scott in Smith Kettewell Eye Research Center used Botulinum toxin (BTX) in human to treat strabismus. In 1989, US-FDA approved BTX for the treatment of strabismus, belpharospasm and hemifacial spasm in patients younger than 12 years old. In 2001, the United Kingdom approved Botox for axillary hyperhidrosis and Canada approach Botox for axillary hyperhidrosis, focal muscle spasticity, and cosmetic treatment of wrinkles at the brow line.^1^

 In July 2004, US-FDA approved Botox for treatment of severe primary axillary hyperhidrosis. It has not been approved by the US-FDA for chronic pain except for chronic migraine. The mechanism of action is by binding presynoptically to the site of anticholinergic nerve terminals and decreasing the release of acetyl-choline and blocking the neuromuscular junction recovery occurring by formation of a new neuromuscular function.^2^ In fact, BTX can cleave snap-25, a presynaptic protamine, on membrane which is required for fusion of neuro-transmitter containing vesicles.^3^ Clinically, 1-u of Botox is approximately equivalent to 3-u of bysport.^4^ In one animal study, Botox had the lowest specific activity, while xermine had the highest specific activity.^5^

## CASE REPORT

A 22 years old man who was a medical student and had complain of sever axillary hyperhidrosis, while topical care had failed in his treatment. Regularly, every 3 to 4 months, he was admitted for treatment of axillary hyperhidrosis with 150 IU Botox for bilateral injection in both axillary regions. He received his first 3 injections without any complications. Each time, three 50-unit vial of Botox was injected in the axillary region bilaterally with complete response and best satisfaction of the results. The last time, instead of three 50-unit vial, in advertently, he received three 100-unit vial of Botox. After about two weeks of injection, had complain of general weakness and fatigue after an extremely exercise session. The next day, he complained of dysphagia to solid foods and choking sensation at night. Type of botulinum toxin-target site has been demonstrated in Table 1.

**Table 1:** Type of botulinum toxin-target site

**Type**	**Target**	**Discoverer**	**Year discovered**
A	Snap-25	Landman	1904
B	Vamp	Ermengm	1897
C1	Syntoxin	Bengston and Seldon	1922
D	Vamp	Robinson	1929
E	Snap -25	Gunnison	1936
F	Vamp	Moller and Schelb	1960
G	Vamp	Gimenz and Ciccarelli	1970

Then had complained of visual problems including diplopia and visual field disturbance. He visited an ophthalmologist and all of the exams included perimetry that was normal. Due to progressive dysphagia and sore throat and previous history of hypersensitivity, he realized that his dysphagia may be related to hypersensitivity; so he visited an asthma and allergy specialist. The physician after performing EKG-CXR which were normal reached to the diagnosis of eosinophilic esophagitis and referred the patientto agastroenterologist for more evaluation. Multiple biopsies of esophagus were undertaken for definite diagnosis of eosinophilic esophagitis. After doing endoscopy and biopsy, all of the reports and results of pathology examination were found to be normal. Due to continuity of his problem especially fatigue and general weakness, he came to our visit and explained the history and asked for more consultant.

According to his history and clinical examination, bilateral mild ptosis and fatigue were noted in his face and a generalized weakness was visible in his muscle. There was a fault in injection of three 100-unit vials of Botox instead of three 50-unit vials. Our clinical diagnosis was complications of Botox injection. For better evaluation, the patient was referred to a neurologist with complete history. The diagnosis of neurologists was the same as ours defined as generalized complications of Botox and treatment was started for the patient prescribing pyridostigmine. Initial response was very good in relief of general weakness and fatigue. The dysphagia was continent after one month of treatment but complete relief of patient’s symptoms occurred after three months. 

## DISCUSSION

Therapeutic uses of botulinum toxin injection are focal dystonia, spasticity, non-dystonic disorders of involuntary muscle activity, strabismus, chronic pain and disorders of localized muscle spasm, smooth muscle hyper active disorders, cosmetic, sweating salivary and allergy disorders.^6^^-^^8^ History taking and physical examination can provide clues about the possibility of botulism, so we should explore the source of the disease as described before.^9 ^The clinical findings of botulism are dry mouth, diplopia, dilated pupil, droopy eyes, droopy face, diminished gag-reflex, dysphagia, dysarthria, dysphonia, difficulty in lifting head, descending paralysis, and diaphragmatic paralysis.^9^

Although dilated pupils and poor papillary contraction to light and drymouth are common in botulism, they are not general in myasthenia gravis. Tensilon test may help to the examiner for diagnosing botulism, although it cannot be differentiated from myasthenia gravis.^10^^,^^11^ In blood test, the standard criterion is a mouth toxicity assay. The other test is neurophysiologic test. ELISA test has become available for rapid screening. Recently, Endopep-Ms following mass spectroscopy (endopeptidas cleavage) has been developed and may be considered as a new criterion.^12^ Electrophysiological findings in botulism may not be present early in the disease and typical findings in EMG are (i) CMAP amplitude in at least 2 muscles; (ii) At least 20% facilitation of CMAP amplitude during tetanic stimulation; (iii) Persistence facilitation for at least 2 minutes after activation, and (iv) No post-activation exhaustion.

The first finding is not seen in all patients with botulism.^13^ If all 4 findings are present, only hypermagnesemia would be in the differential diagnosis and can quickly be approved by blood magnesium level.^14^ Pyridostigmine (mestinon) is a reversible acetyl-choline esterase inhibitor that US-FDA has approved for treatment of myasthenia gravis. It is also used off table for emergency in reversal of non-depolarizing neuromuscular junction blockers during anesthesia. The neurologists prescribed 60 mg of pyridostigmine, three times a day and our patient experienced a rapid relief for most of the symptoms except for dysphagia which was continued. The most common side effects of mestinon are cholinergic such as stomach cramps and diarrhea and generally are considered minor.^15^ As most of the side effects of Botox have been reported in other therapeutic indications except aesthetic and hyperhidrosis, we reported this case to be about the dangerous side effects that may occur in aesthetic and hyper-hyperhidrosis use of Botox that all of us should be familiar with the side effects and treatment of the complication.

## CONFLICT OF INTEREST

The authors declare no conflict of interest.
